# Ketamine induces apical extracellular matrix modifications in *Caenorhabditis elegans*

**DOI:** 10.1038/s41598-022-24632-5

**Published:** 2022-12-21

**Authors:** Duygu Yücel

**Affiliations:** Genome and Stem Cell Center (GENKOK), Erciyes University, 38039 Melikgazi, Kayseri Turkey

**Keywords:** Neuroscience, Genetics, Developmental biology, Experimental organisms

## Abstract

Ketamine is a widely used anesthetic agent since 1960s and has recently been exploited for its rapid antidepressant action at subanesthetic doses. It has been demonstrated that ketamine induces alterations in extracellular matrix (ECM) in rodent models which in part plays a role in its anti-depressant action. The nematode *Caenorhabditis elegans* serves as a powerful tool for understanding mechanisms of drug action with its short life cycle, genetic amenability and conserved cellular processes. Further investigation is required particularly in in vivo systems to gain broader understanding of ketamine’s actions. In this study, we aimed to decipher ketamine-mediated alterations using *C. elegans* as a model. We show that ketamine specifically induces apical extracellular matrix modifications (aECM) in the vulva and the cuticle. Ketamine treatment phenocopies neuronal migration and vulval invagination defects of chondroitin mutants despite wild-type like chondroitin staining pattern. Normal vulval expansion and defective vulval eversion phenotypes of ketamine-treated animals are suggestive of alterations in the network of aECM factors which do not impinge on chondroitin. Ketamine ameliorates impaired movement of a group of *roller* mutants characterised with collagen defects in the cuticle and RNA-seq identifies that 30% of the cuticular collagens are upregulated in response to ketamine. Ketamine alters aECM, neuronal migration and collagen expression in *C. elegans*. We propose *C. elegans* as a putative animal model to investigate ketamine-mediated ECM modifications.

## Introduction

Ketamine is a widely used anesthetic agent since 1960s and has recently been exploited for its rapid antidepressant action^1–3^. N-methyl-d-aspartate (NMDA) receptor blockade is the underlying mechanism for anesthetic action of ketamine, however how ketamine exerts its antidepressant effect is complex and still not clear. Increased synaptogenesis after low-dose ketamine treatment via modulation of extracellular matrix (ECM) has been proposed as one of the mechanisms of ketamine’s antidepressant action which is sustained long after the drug is metabolised^4,5^. It has been previously demonstrated that ketamine induces epithelial phenotypic changes (EPC) and alterations in ECM in animal and cell line models^5–10^. The nematode* Caenorhabditis elegans* serves as a powerful tool for understanding mechanisms of drug action with its short life cycle, genetic amenability and conserved cellular processes. *C. elegans* has a simple anatomy and fully characterised, invariant cell lineage. A wild-type animal has 959 somatic cells which are traceable from zygote to adult stage. The transparent body of *C. elegans* consists of wide variety of epithelial tubes despite only 959 somatic cells. The tubular structures such as cuticle and the vulva are lined with extracellular matrices composed of collagens, lipids, chondroitin and heparan glycosaminoglycans (GAG) some of which are shared with the mammalian ECM components^11^.

One of the widely investigated tissue in *C. elegans* is vulva which is generated via orchestration of mainly EGFR/Ras/MAPK and Notch signalling. The egg-laying apparatus vulva in the hermaphrodite *C. elegans* is comprised of 22 cells which are derived from epithelial precursor cells through three rounds of cell divisions within 5 h^12^. More than hundred mutants isolated through mutagenesis screens were identified on the basis of vulval lineage defects, and were broadly classified as being vulvaless (vul) and multivulva (muv)^12–15^. Vulval morphogenesis can also be disrupted without a cell lineage defect. One such vulval phenotype arises due to defective epithelial invagination causing a misshaped vulva with a squashed vulval lumen appearance termed as Sqv. Disruption of the chondroitin biosynthesis has been reported to account for formation of Sqv phenotype on the basis of *sqv* mutants carrying mutations in genes which encode components of chondroitin biosynthesis.

In this study, we aimed to decipher ketamine-mediated modifications using *C. elegans* as a model. We show that ketamine specifically induces apical extracellular matrix modifications (aECM) in the vulva and the cuticle. Ketamine treatment phenocopies neuronal migration and vulval invagination defects of chondroitin mutants despite wild-type like chondroitin staining pattern. Normal vulval expansion and defective vulval eversion phenotypes of ketamine-treated animals are suggestive of alterations in the network of aECM factors which do not impinge on chondroitin. Ketamine ameliorates impaired movement of a group of *roller* mutants characterised with collagen defects in the cuticle and RNA-seq identifies that 30% of the cuticular collagens are upregulated in response to ketamine. Our findings identify BLIMP-1/BLMP-1 as a putative novel molecular target for ECM modulatory effects of ketamine. We propose that ketamine modulates ECM components in part via orchestration of Wnt and TGF-beta signalling pathways and regulatory network of transcription factor BLMP-1.

## Results

### Ketamine-treated animals show stochastic development with slower feeding rate

**Figure 1:**
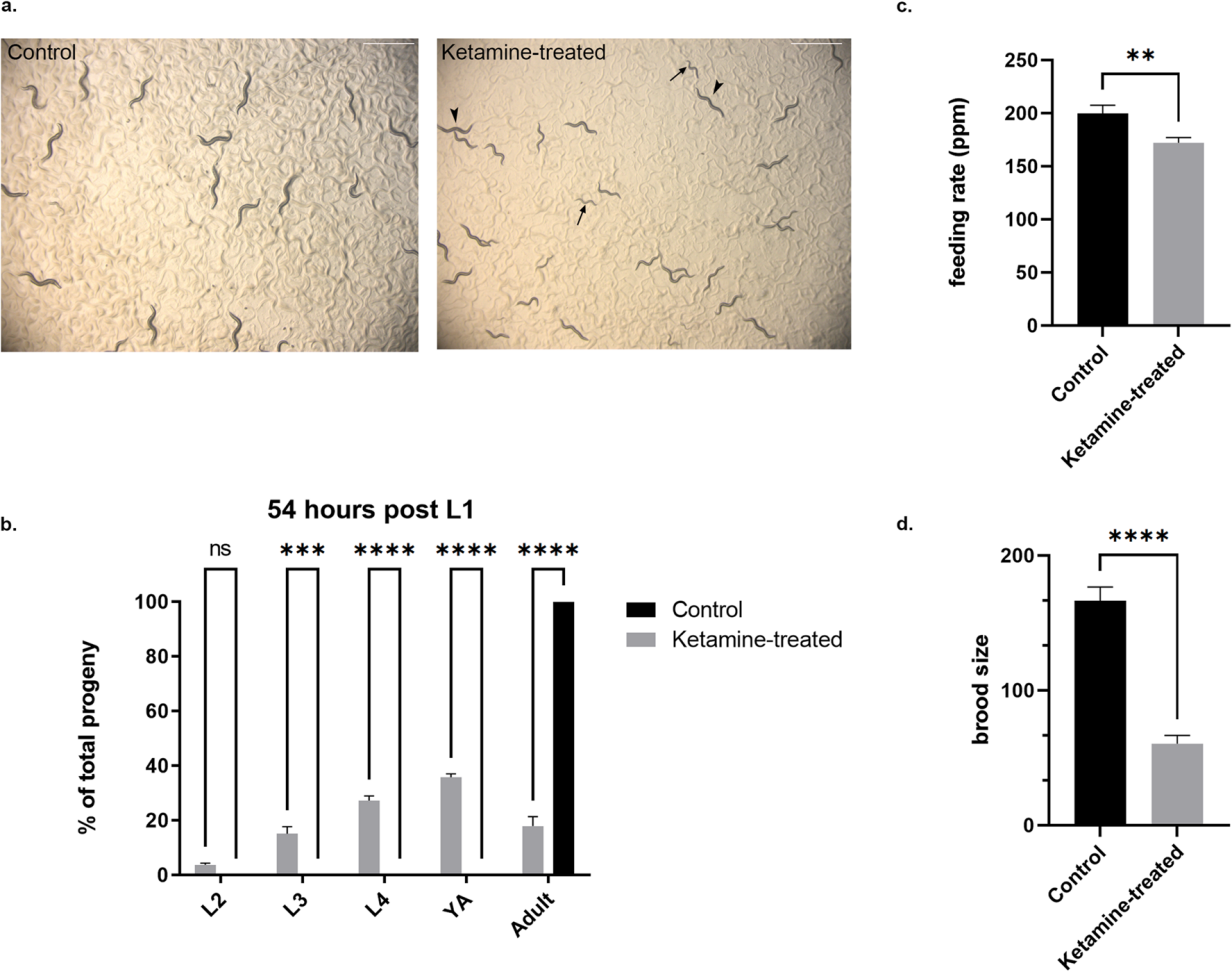
Ketamine-treated animals display stochastic development with slower pumping rate and reduced brood size (**a**) Control and ketamine-treated wild-type animals at 54 h post L1 is shown. In the control plate, all animals reach adulthood 54 h post L1 stage whereas in ketamine-treated plate mixed stage animals were observed as indicated with arrows showing L2 stage animals and arrowheads adults. Scale bar indicates 1 mm. (**b**) Worms grown in 2.5 mM ketamine (n = 1036) showed stochastic growth consisting of L2, L3, L4, young adult and egg-laying adult at 54 h post L1 whereas 100% of the control animals (n = 958) reached egg-laying adult stage. (Two-way ANOVA with Šídák's multiple comparisons test, mean ± sem, ***p < 0.001, ****p < 0.0001) (**c**) pharyngeal pumping rate per minute (ppm) was measured for control (n = 26) and ketamine-treated animals (n = 26), *p* = 0.0048. (Student’s *t*-test, unpaired, mean ± sem, ** *p* < 0.01) (**d**) The brood size of control (n = 30) and ketamine-treated animals (n = 25) were measured until egg-laying ceased. (Student’s *t*-test, unpaired, mean ± sem, ****p  < 0.0001).

*Caenorhabditis elegans* has a rapid life-cycle. After hatching, it becomes an egg-laying adult within 2.5 days at 25 °C. We have monitored control and ketamine-treated animals for 48–72 h. At the 54-h mark, all animals in the control plate were adults whereas only a small fraction of ketamine-treated animals could reach adulthood (Fig. 1a). As shown in Fig. 1b, at 54 h post-L1 stage, while 100% of the control plate (n = 652) contained egg-laying adults, only 18% ± 3.4 of the ketamine-treated animals reached adulthood (n = 598) (Two-way ANOVA, mean ± sem, Šídák's multiple comparisons test, *p* < 0.0001). At this stage, ketamine-treated plate contained 4% ± 0.6 of L2 stage, 15% ± 2.6 of L3, 27% ± 1.6 of L4 and 36% ± 1.2 of young adult animals. Given that ketamine works as an anesthetic, we tested whether developmental delay might be due to slow pharyngeal pumping rate. The pharynx of the worm works as a neuromuscular pump where proper pharyngeal movements are required for a normal feeding. Feeding quality can be measured as a read-out of pharyngeal pumping rate per minute (ppm). Feeding rate of ketamine-treated and no-treatment control animals were measured in young adults (Fig. 1C). The feeding rate in ketamine treated animals was 15% slower than the wild-type animals (Student’s t-test, unpaired, *p* = 0.0048). Although the difference was subtle it reached significance levels. In order to explore whether abnormalities caused by ketamine extends to reproduction process, the brood size was determined for control and ketamine treated animals. The average brood number in ketamine-treated animals decreased by 63% as compared to control animals (Fig. 1d).

### Ketamine disrupts vulval invagination without changing vulval cell fates

**Figure 2:**
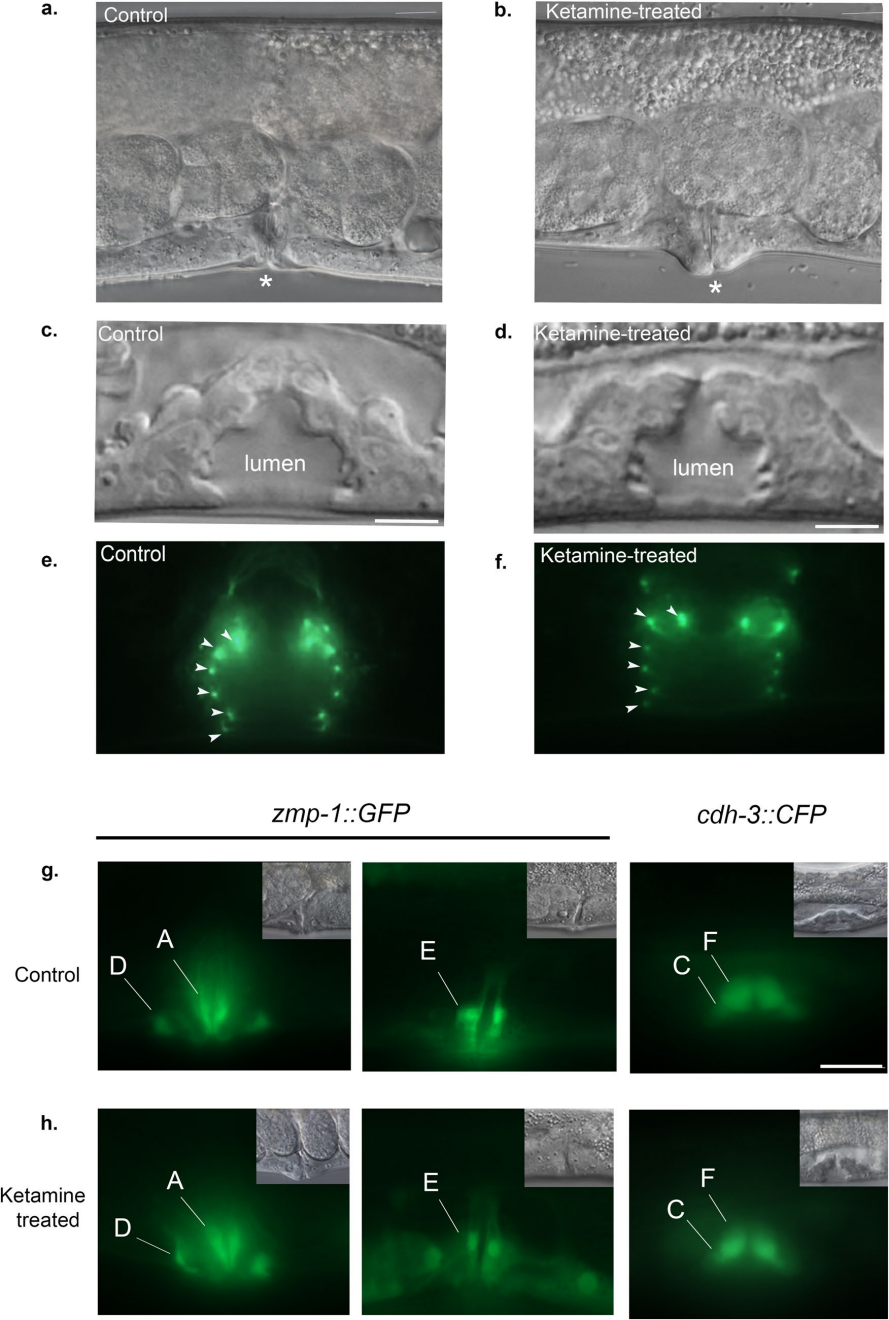
Ketamine disrupts vulval invagination without changing vulval cell fates (**a**) Control and (**b**) ketamine-treated animals at adult stage are depicted. The penetrance of Sqv phenotype is 98% (n = 583 for control and n = 569 for ketamine-treated). Vulva is marked with an asterisk. (**c**) Vulval toroids are assessed using *ajm-1::GFP* adherens junction marker. Nomarski images of animals at L4 stage are shown for control and (**d**) ketamine-treated animals. Vulval extracellular space is marked as “lumen”. (**e**) *ajm-1::GFP* marker is expressed between the toroid structures. Seven toroids were detected for both control and (**f**) ketamine-treated animals. Arrowheads indicate adherens junctions marked with GFP. Six out of seven toroids are visible in the focal plane. (**g**) Vulval cell fates were examined in control and in (**h**) ketamine-treated animals using *zmp-1::GFP* marker at adult stage and *cdh-3::CFP* marker at L4 stage. Ketamine-treated animals did not display cell fate specification defects. Vulval cells were intact in ketamine-treated animals. VulA, VulD and VulE were visualized using *zmp-1::GFP* marker. VulC and VulF were visualized using *cdh-3::CFP*. Scale bar indicates 10 μm for all panels. Anterior is to the left, dorsal is up.

*Caenorhabditis elegans* vulva is a tube-like structure generated from Ras-dependent and Notch-dependent vulval precursor cells. In the final round of divisions, a group of vulval cells detach from the cuticle and starts to invaginate. The migration of specific subset of vulval cells from epithelial plane allows formation of vulval tube termed as lumen by creating an invagination space. Animals with defective vulval invagination has been reported to carry mutations in genes encoding for chondroitin synthesis^16,17^. In severe cases, this vulval phenotype can be identified with its narrow lumen at L4 stage. At adult stage, when the animals are filled with eggs, vulva protrudes abnormally^17^. Due to narrow or squashed appearance of the vulval lumen, this phenotype is termed as squashed vulva, shortly Sqv. We observed that ketamine treatment causes a partial collapse of the vulval invagination resulting in a Sqv phenotype at adult stage with a penetrance of 98% (n = 583 for control and n = 569 for ketamine-treated). (Fig. 2b). Wild-type animal’s vulva is shown in the control panel of Fig. 2a,c, at adult stage and L4 stage, respectively. In ketamine-treated animals, vulva protruded abnormally. The vulval extracellular space marked “lumen” in Fig. 2c,d, resembles a “Christmas tree” indicative of a mid-L4 stage^18^. The vulva lumen is filled with apical extracellular matrix structures composed of mainly chondroitin proteoglycans, ZP domain proteins and other glycoproteins which mediates luminal expansion and vulval eversion^19^. At L4 stage, ketamine-treated animals had a wild-type like vulval lumen (Fig. 2b,d). The Sqv phenotype was visible at adult stage. We further analysed the vulval integrity of ketamine-treated animals using *ajm-1::GFP* reporter which is an adherens junction marker expressed between the vulval toroids that are formed at L4 stage. Vulval toroids are formed through cell fusion and toroid structures identified in ketamine-treated animals were similar to control animals. In both control and ketamine-treated animals, seven vulval toroids were identified (Fig. 2e,f). There was no obvious vulval lumen or toroid morphogenesis defect in ketamine-treated animals.

**Figure 3:**
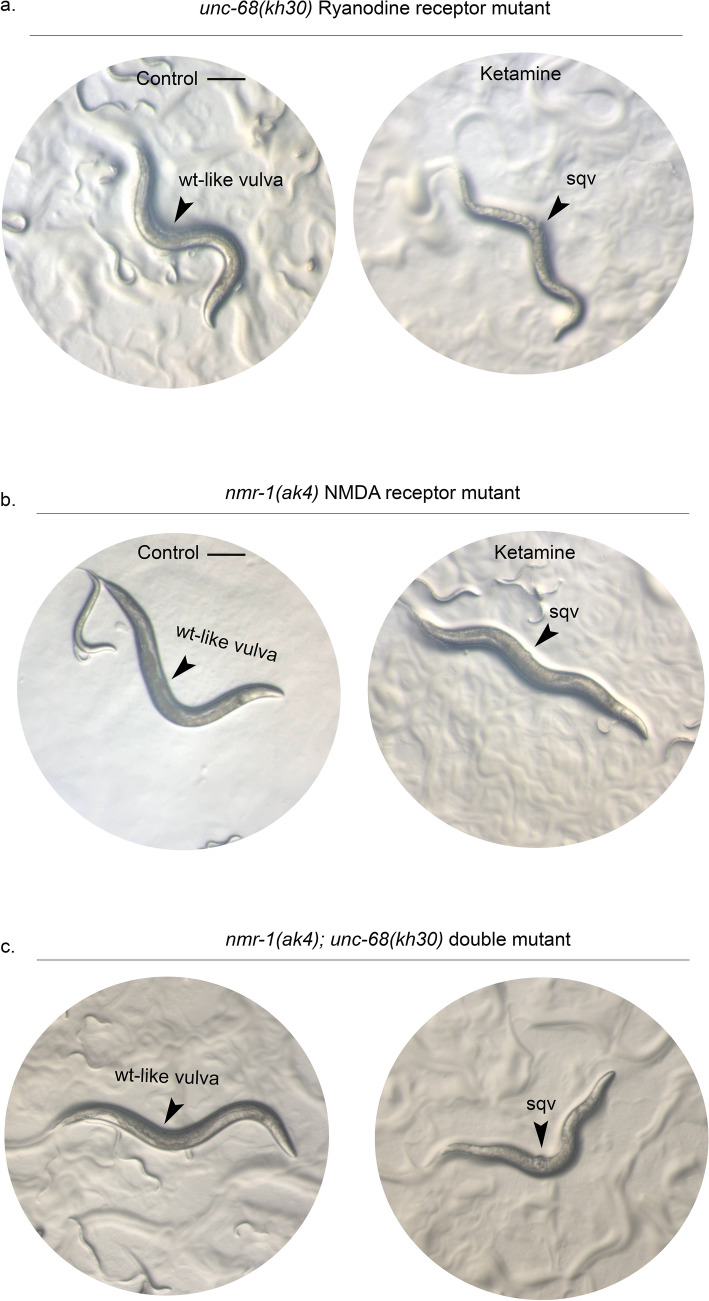
Ketamine receptor mutants display sqv phenotype (**a**) Worms carrying a mutation in ryanodine receptor *unc-68* (n = 84) or (**b**) in NMDA receptor *nmr-1* (n = 95) both show vulval invagination defect (Sqv phenotype) when exposed to ketamine. (**c**) Double mutants (n = 76) showed a similar penetrance for Sqv phenotype as single mutants. Arrowheads indicate the vulva. Scale bar 200 μm.

We then examined vulval cell fate differentiation using vulval reporter markers *zmp-1::GFP* and *cdh-3::CFP*; *ceh-2::YFP* reporter markers (Fig. 2g). *zmp-1::GFP* is expressed in VulA, VulD and VulE at adult stage. 100% of the ketamine-treated animals (n = 82) displayed correct expression of *zmp-1::GFP* reporter similar to control animals (n = 63). VulB, VulC and VulF were visualised using *cdh-3::CFP; ceh-2::YFP* reporter in L4-stage animals where ketamine-treated animals (n = 102) showed no obvious expression defects as compared with the controls (n = 84). Figure 2h shows that ketamine-treated animals have intact vulval cells. VulB is not shown in the representative images but was assessed under the microscope and found intact for ketamine-treated animals. Having confirmed that ketamine-treated animals show vulval invagination defect without a vulval cell differentiation abnormality, animals carrying mutations in known ketamine receptors in the worm, that is RyR and NMDA receptors, namely *unc-68* and *nmr-1* were tested for formation of Sqv phenotype^20,21^ (Fig. 3). We have observed that *unc-68(kh30)*, *nmr-1(ak4)* and *nmr-1(ak4); unc-68(kh30)* mutant animals show highly penetrant Sqv phenotype clearly identified under a dissecting microscope. For *unc-68* mutants (Fig. 3a), 84 animals were scored 98% of which displayed Sqv phenotype and for *nmr-1* mutants (Fig. 3b), 95 animals were examined where 99% of the animals had a Svq phenotype. Double mutants (n = 76) showed a similar penetrance for Sqv phenotype (98%) as single mutants (Fig. 3c).

### Ketamine-treated animals display HSN migration defect at adult stage

**Figure 4:**
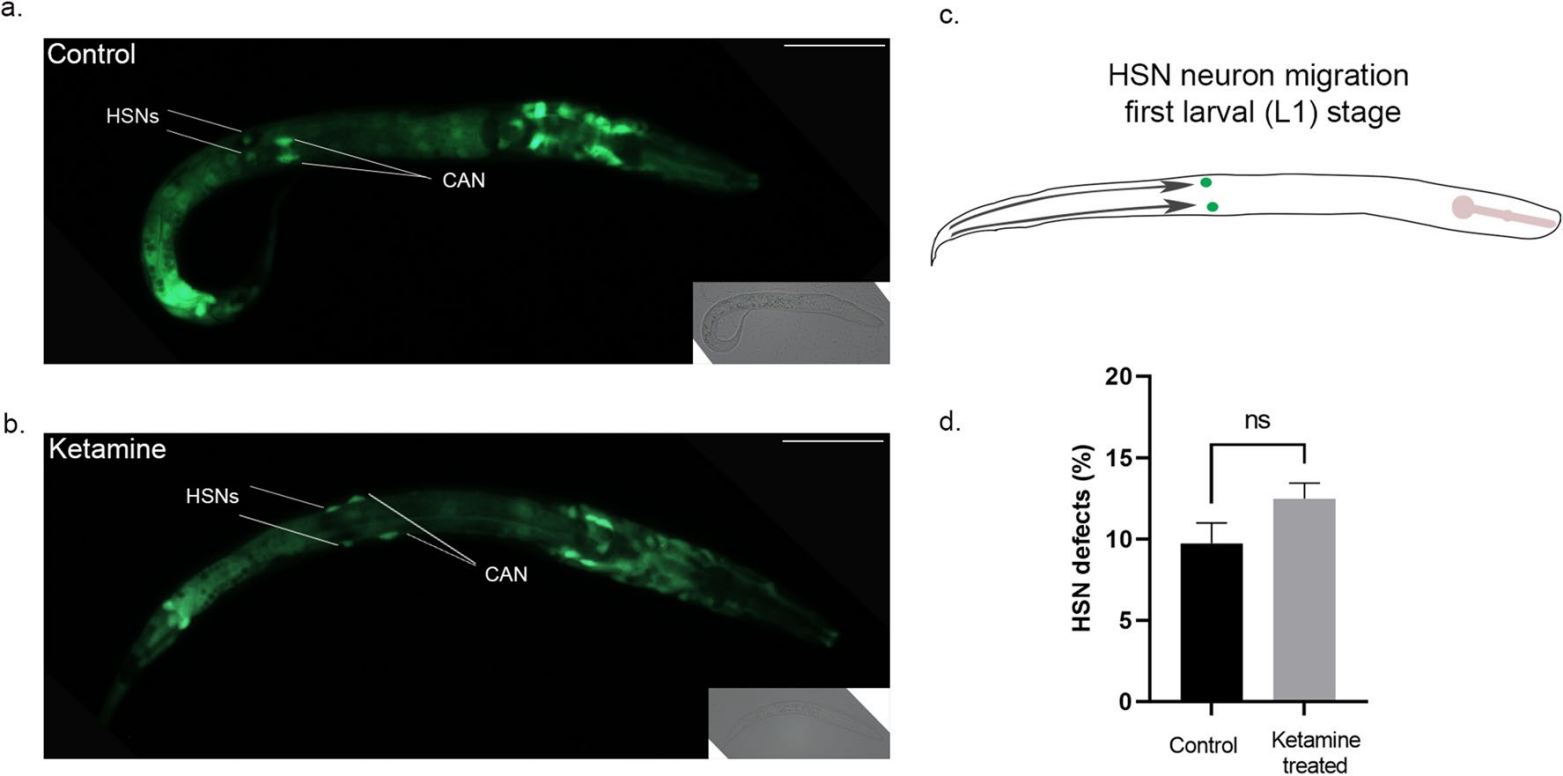
HSN cell bodies migrate to the correct body position in ketamine-treated animals at first larval stage (**a**) Control animal at first larval stage (L1) expressing *kal-1::GFP* reporter marker in hermaphrodite specific neurons (HSN) and Canal Associated Neurons (CAN) is shown (**b**) Ketamine-treated animals at L1 stage expressing *kal-1::GFP* reporter do not show HSN migration defects (**c**) Illustration of HSN migration at first larval (L1) stage. HSNs are born in the tail during embryogenesis and migrate towards midbody of the worm (**d**) Migration defects of control (n = 172) and ketamine-treated animals (n = 156) were compared. Four biological replicates were used. (Student’s *t*-test, paired, mean ± sem, *p* = 0.2883; ns: not significant) Scale bar 25 μm. Anterior is to the right and dorsal is up.

Disruption of vulval morphogenesis without a cell lineage defect in ketamine-treated animals is similar to phenotypes displayed by *sqv* mutants. In addition to their vulval abnormality, *sqv* mutants were reported to display migration defects^22,23^. We tested whether ketamine-treated animals phenocopy *sqv* mutants’ migration defects and to do this, we tracked the migration of Hermaphrodite Specific Neurons (HSN). HSNs are bilaterally symmetric serotonergic motor neurons which control egg laying in the hermaphrodite animals. HSNs are born in the tail during embryogenesis and migrate anteriorly towards the middle of the embryo (Fig. 4c)^24,25^. Axonal outgrowth which is completed by young adult stage begins in L2 stage where HSNs become polarised ventrally. We first checked migration of HSN neurons at L1 stage worms using *kal-1::GFP* marker (Fig. 4). Ketamine-treated animals were analysed for migration defects of bilateral HSN neurons at first larval stage. Migration of HSNs at L1 stage ketamine-treated animals (Fig. 4a) was similar to no treatment controls (Fig. 4b,c, Student’s *t*-test, paired, mean ± sem, *p* = 0.2883).

**Figure 5:**
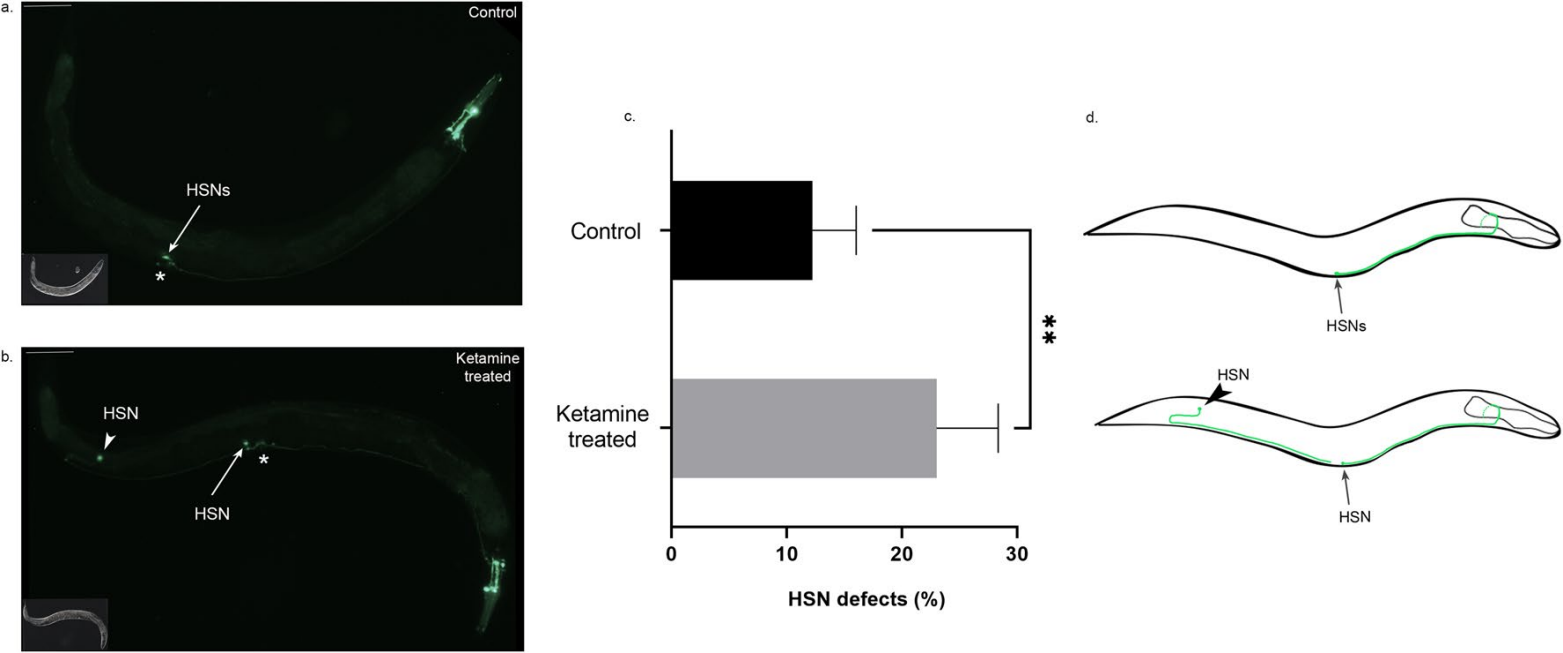
Ketamine-treated animals display HSN migration defect at adult stage (**a**) In control animal expressing *tph-1*::GFP at adult stage, HSN cell bodies marked with an arrow migrate to the midbody being positioned posterior to the vulva. (**b**) In ketamine-treated animals, one of the HSNs fail to migrate to its correct body position. HSN with a migration defect is shown with an arrowhead. (**c**) Migration defects of control (n = 252) and ketamine-treated animals (n = 258) were compared. Four biological replicates were used. (Student’s *t*-test, paired, mean ± sem, *p* = 0.0055; **p < 0.01). (**d**) Illustration of HSN migration at adult stage. HSN cell bodies are located posteriorly near the vulva in a wild-type animal. In ketamine-treated animals, one of the HSN cell body fails to migrate to its correct position. Asterisk indicates vulva. Scale bar 75 μm. Anterior is to the right.

Each HSN neuron sends a single axon which stereotypically extend towards ventral position and around the vulva^26^. HSN axon then enters the ventral nerve cord and grow anteriorly towards the head. Having confirmed that HSNs display normal migration at early larval stage in ketamine-treated condition, the migration and axonal guidance of HSNs were further analysed at adult stage where axonal growth of HSNs is completed. Using a different reporter that is *tph-1::GFP* marker, adult worms were treated with ketamine and their HSN migration and axonal guidance was examined. As shown in Fig. 5a, in control animals HSN cell bodies are positioned correctly at the vulva extending their axon anteriorly reaching the head. Compared with the control animals, in ketamine-treated animals (Fig. 5b) one of the bilateral HSNs was mispositioned at the tail failing to migrate to its correct position. No obvious axonal defects were observed. The incidence of HSN migration defects in ketamine-treated animals was 23% (Fig. 5c,d, Student’s *t*-test, paired, mean ± sem, *p* = 0.0055; **p < 0.01).

### Ketamine-treated animals show wild type-like chondroitin phenotypes

**Figure 6:**
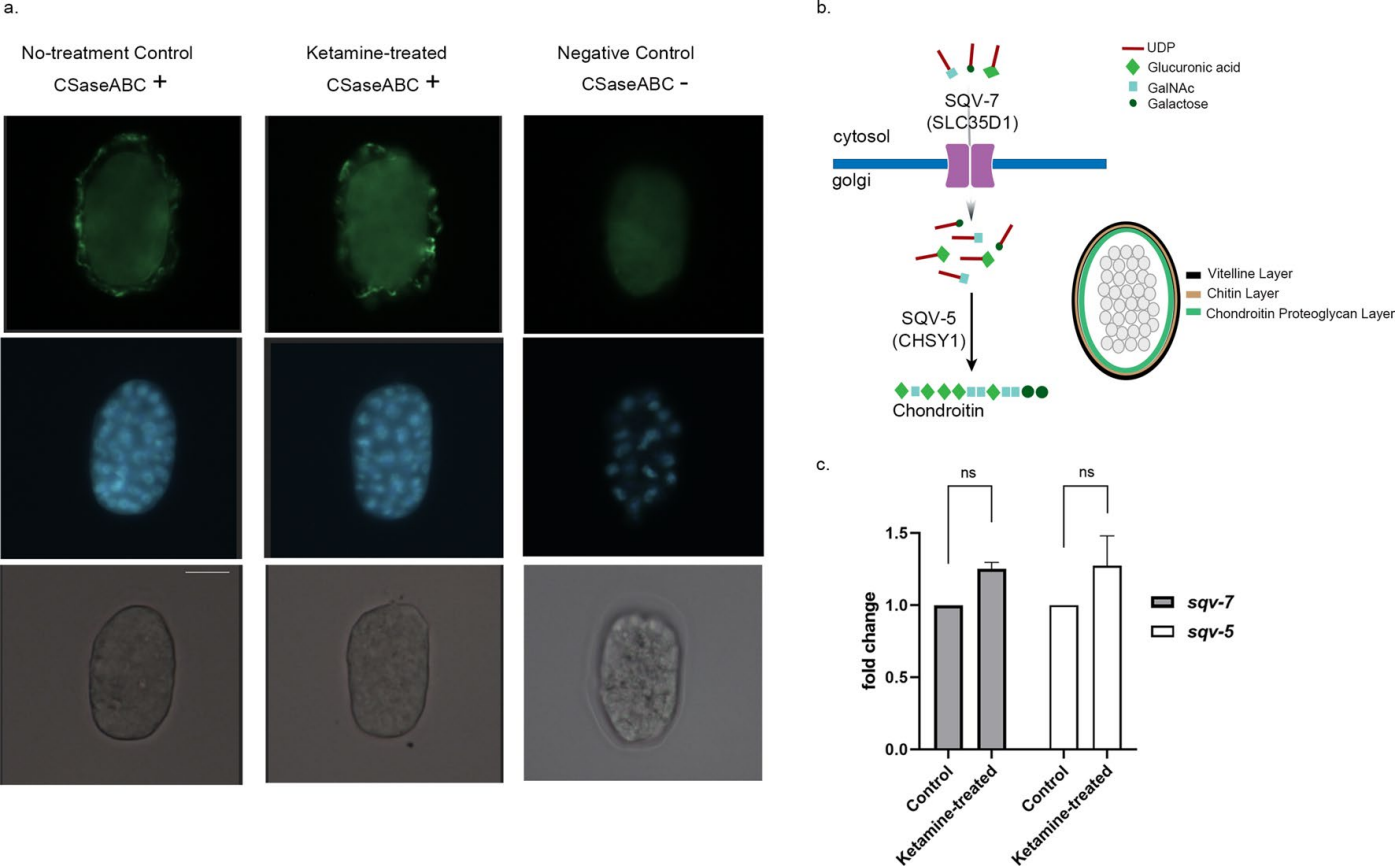
Ketamine-treated animals show wild type-like chondroitin phenotypes (**a**) Control and ketamine-treated embryos stained with anti-chondroitin antibody (anti-proteoglycan ΔDi-0S antibody 1B5) are shown in the top panel. Chondroitinase ABC (CSase ABC) enzyme was used for no-treatment and ketamine-treated embryos. As per negative control no enzyme was used. Middle panel shows the nuclei of the embryo stained with DAPI and the bottom panel shows DIC. Scale bar 25 μm (**b**) The organization of the eggshell is depicted. Chondroitin proteoglycan layer resides beneath the vitelline and chitin layers. The biosynthesis of the chondroitin chains is illustrated with a focus on two key regulatory proteins, UDP-sugar transporter SQV-7 and chondroitin synthase SQV-5 with mammalian homologs of SLC35D1 and CHSY1, respectively. (**c**) Relative *sqv-5* and *sqv-7* expression levels in control and ketamine-treated animals were measured using real time RT-PCR. Data shown are derived from three biological replicates (Two-way ANOVA with Šídák's multiple comparisons test, mean ± sem, *p* = 0.3032 for *sqv-7* and *p* = 0.2588 for *sqv-5*, ns: not significant).

Given that ketamine-treated worms display Sqv phenotype that is characteristic of animals carrying mutations in the genes encoding for chondroitin synthesis pathway components such as the chondroitin synthase *sqv-5* (squashed vulva-5) mutants and uridine 5′-diphosphate–sugar transporter *sqv-7* mutants; we embarked on assessment of chondroitin abnormalities in ketamine-treated animals^22,23^. To do this, an antibody specific for Chondroitin-0-sulphate was used which detects chondroitin stubs digested with chondroitinase ABC (CSase ABC) enzyme. Embryos of the ketamine-treated adults were examined for their chondroitin proteoglycan (CPG) layer morphology. The eggshell of the *C. elegans* is composed of three outer layers with CPG layer being defined only a decade ago (Fig. 6b)^27^. The CPG layer in ketamine-treated animals was intact as observed in no-treatment control (Fig. 6a, top panel). Negative control embryos where no CSase ABC was used showed no staining indicative of the specificity of the staining for chondroitin stubs. When integrity of the CPG layer is disrupted, the eggshell display ruptured morphology. In ketamine-treated condition, the eggshell integrity was reserved. In *C. elegans* the biosynthetic machinery for chondroitin proteoglycan synthesis is conserved yet less complex compared to mammals^28^. The key regulatory components for chondroitin synthesis is transport of UDP-sugars by SQV-7 and elongation of chondroitin chains by SQV-5 (Fig. 6b). In order to further assess chondroitin abnormalities, transcript levels of *sqv-5* and *sqv-7* were quantified using real-time RT-PCR (Fig. 6c). The expression levels of *sqv-5* and *sqv-7* showed no significant difference compared with the no-treatment control (Two-way ANOVA with Šídák's multiple comparisons test, mean ± sem, *p* = 0.3032 for *sqv-7* and *p* = 0.2588 for *sqv-5*).

### Ketamine modulates cuticle extracellular matrix

**Figure 7:**
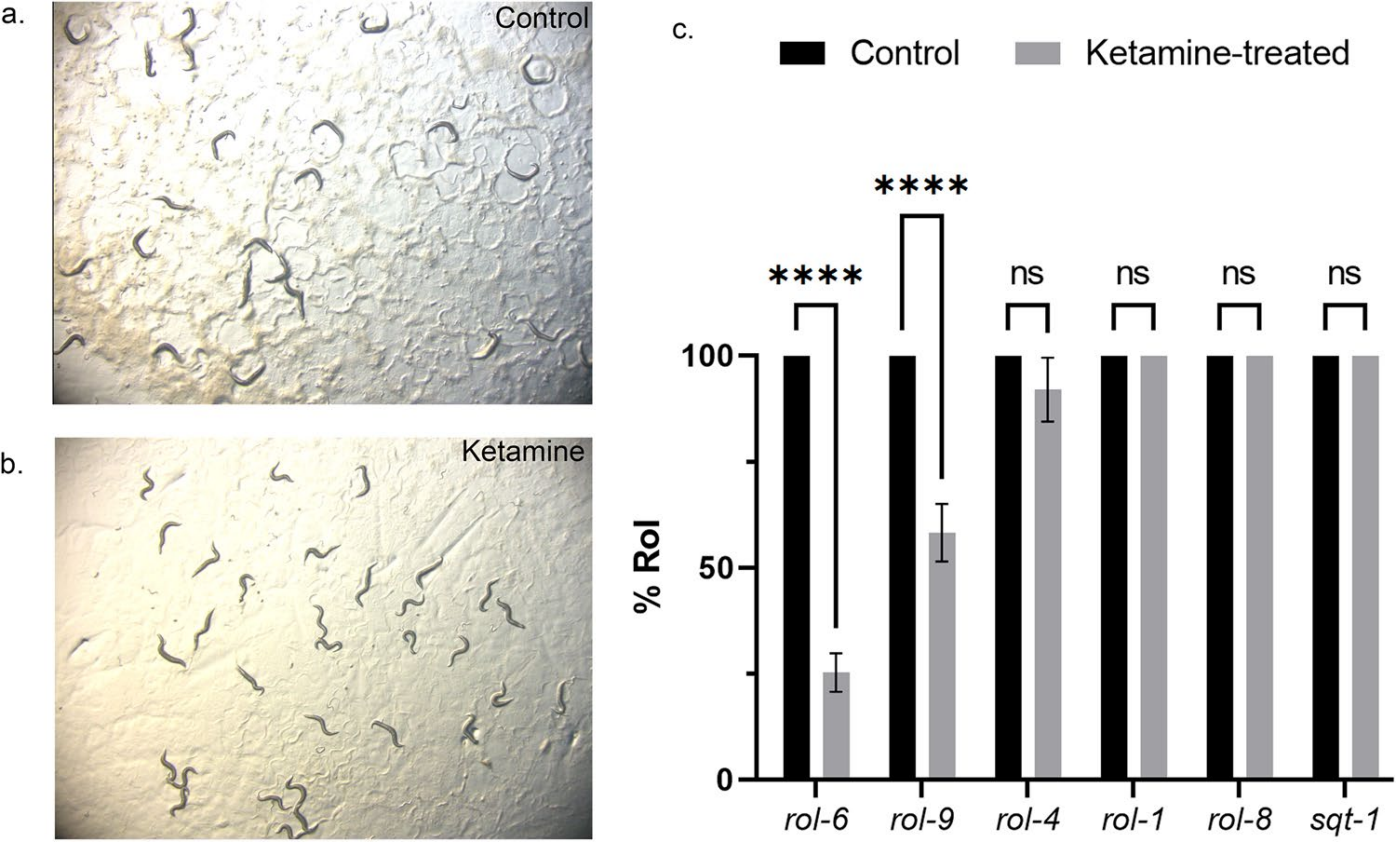
Ketamine-treatment ameliorates the roller phenotype of collagen mutants (**a**) *rol-6(e187)* mutant with circular movement pattern is shown (**b**) Ketamine-treatment ameliorated the movement defect of *rol-6(e187)* mutants (**c**) Collagen mutants are tested for their roller phenotype suppression upon ketamine-treatment. *rol-6* (n = 295) and *rol-9* (n = 327) mutants’ movement defect was significantly ameliorated with ketamine treatment (Two-way ANOVA with Šídák's multiple comparisons test, mean ± sem, ****p < 0.0001 for both mutants). *rol-1*, *rol-4*, *rol-8*, *sqt-1* collagen mutants continued to display roller phenotype upon ketamine-treatment with a slight decrease in *rol-4* mutants without significant changes (*p* = 0.0588 for *rol-4* and *p* > 0.9999 for the rest, ns: not significant).

Ketamine-treated animals display Sqv phenotype characteristic of chondroitin mutants yet have wild-type like chondroitin levels and distribution. Sqv phenotype arises due to disruption of vulval lumen formation which is coordinated via apical extracellular matrix components including chondroitin proteoglycans, ZP domain proteins and other glycoproteins. The eggshell is also formed through aECM components and ketamine-treated animals did not show an obvious eggshell rupture as evidenced with the staining of CPG layer against chondroitin stubs. It was curious whether ketamine-treatment modulates cuticle formation which is another tubular structure formed of aECM in the worm. The epidermal cuticle which maintains the body shape is formed of collagen and non-collagen components. Cuticle collagen gene mutants can affect the body shape in various ways one which is twisted body axes termed as a “Roller” phenotype, shortly “*rol*” (Fig. 7a). *rol-6(e187)* mutants depicted in Fig. 7a have a helically twisted cuticle such that they rotate around their long axis moving in a rolling fashion as opposed sinusoidal wave pattern of wild-type worms. When treated with ketamine, *rol-6* mutants’ movement defect was ameliorated with 25% rollers as compared with 100% in untreated *rol-6* mutants (Two-way ANOVA, mean ± sem, Šídák's multiple comparisons test, *p* < 0.0001) (Fig. 7b). Ketamine’s action on apical extracellular complex was further tested using other cuticle collagen mutants (Fig. 7c). Ketamine treatment could ameliorate the Rol phenotype of *rol-9* but not *rol-1*, *rol-4*, *rol-8*, *sqt-1* mutants. There was a slight decrease in the rolling animals in *rol-4* mutants but without significant changes (Two-way ANOVA, Šídák's multiple comparisons test, *p* = 0.0588). For *rol-1*, *rol-8* and *sqt-1* mutants, control and ketamine-treated animals both displayed 100% roller phenotype. The video recordings showing amelioration of Rol phenotype for *rol-6* and *rol-9* mutants are shown in Supplementary Videos 1a, 1b and 2a and 2b.

**Figure 8:**
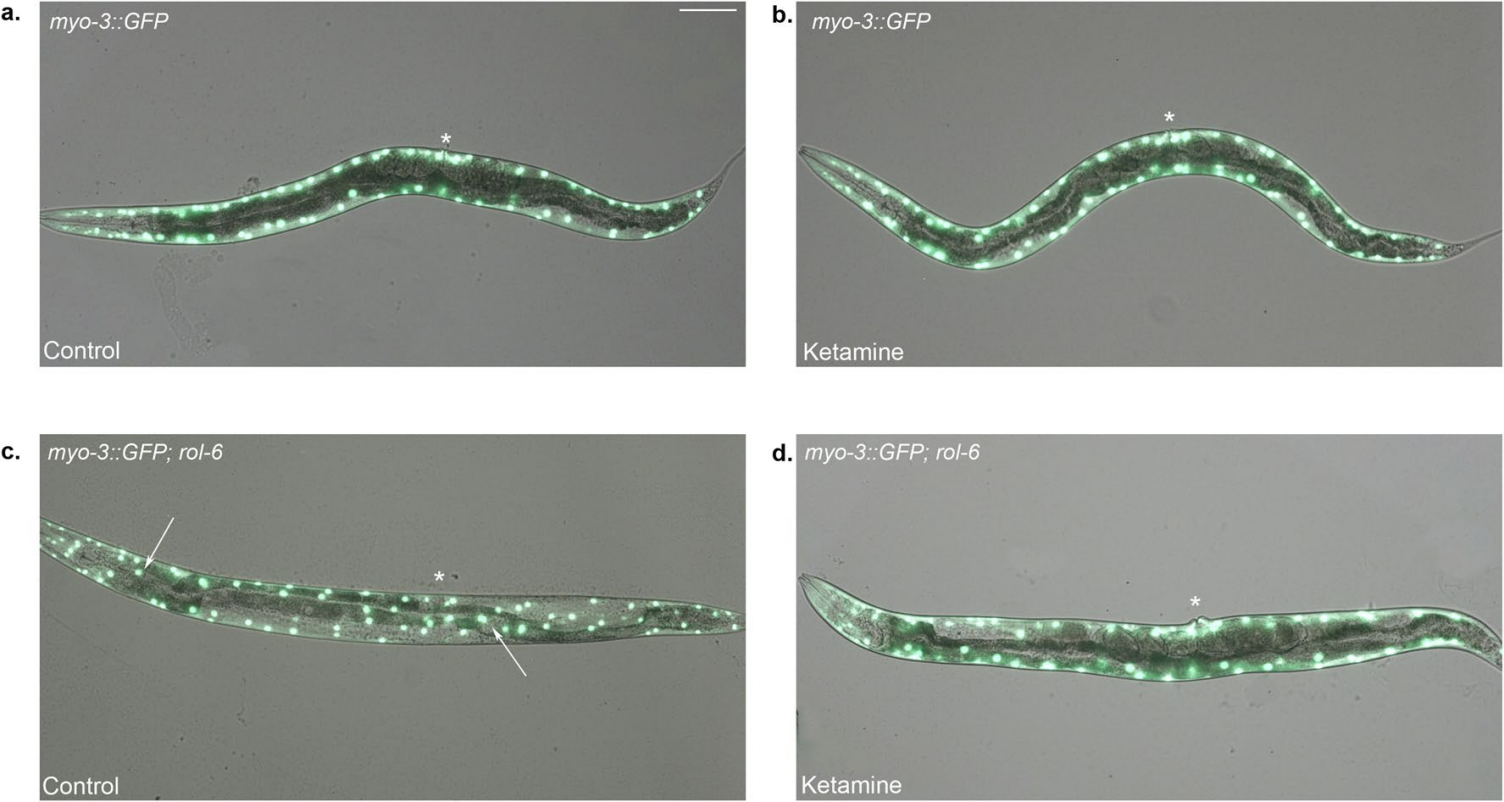
The cuticle structure of the *rol-6* mutants is restored upon ketamine treatment (**a**) Wild-type animal expressing *myo-3::GFP* reporter construct in control and (**b**) ketamine-treated conditions. Body wall muscles are longitudinally oriented along the body of the worm. (**c**) *rol-6(e187)* mutant expressing *myo-3::GFP* reporter construct in control condition is shown. Arrows show the positions of the helical twist in the cuticle structure. (**d**) In ketamine-treated *myo-3::GFP*; *rol-6(e187)* mutant, a wild-type like body wall muscle expression pattern is detected. Vulva is marked with an asterisk. Anterior is to the left. Scale bar shows 75 micron for all panels.

The cuticle and the internal organs of the *roller* mutants are helically twisted. It is possible to visualise the helical cuticle pattern using reporter constructs. In order to assess the cuticle structure of the ketamine-treated wild-type like moving *rol-6* mutants, we used *myo-3::GFP* reporter which is expressed in the body wall muscle cells. These muscle cells are attached to the hypodermis and the cuticle rendering *myo-3::GFP* as a suitable reporter construct to visualize the helically twisted body pattern. In *myo-3::GFP* animals treated with M9 buffer as a vehicle (Fig. 8a) and with ketamine (Fig. 8b), body wall muscles are longitudinally oriented along the body of the worm. In *rol-6* mutants (Fig. 8c), the longitudinal alignment of the body wall muscle cells is disrupted as shown with arrows indicating the position of the helical twist. In ketamine-treated, wild-type like moving *rol-6* mutants, the cuticle structure is restored as depicted in Fig. 8d. *myo-3::GFP* expression pattern for ketamine-treated *rol-9(sc148)* mutant is shown in Supplementary Fig. 1. Similar to *rol-6* mutants, the cuticle structure of the *rol-9* mutants is ameliorated via ketamine exposure.

### Cuticular collagens are upregulated in ketamine-treated wild-type animals

In order to identify the underlying mechanism of ketamine-induced modifications, we performed RNA-seq experiments which showed that ketamine treatment in wild-type animals upregulated 473 genes and downregulated 254 genes (Supplementary Table 1a and 1c). Genes with at least a two-fold difference at an FDR < 0.05 were assigned as differentially expressed. Functional enrichment profiling of the ketamine-responsive upregulated genes has shown “structural constituent of cuticle” (GO:0042302) as the top Gene Ontology (GO) category with 55 genes (Supplementary Table 1b). Top 5 GO category included iron ion binding (GO:0005506), tetrapyrrole binding (GO:0046906), collagen trimer (GO:0005581) and steroid hydroxylase activity (GO:0008395). Tetrapyrrole binding (GO:0046906), iron ion binding (GO:0005506) and steroid hydroxylase activity (GO:0008395) categories had similar set of genes all of which are stress response genes corresponding to cytochrome P450 family. As expected, CYP genes are activated for detoxification of the animal. Cuticle development (GO:0042335) and molting cycle (GO:0042303) were also among the GO list ranked as 7th and 9th, respectively. Cuticular collagens significantly upregulated in ketamine-treated wild-type animals are listed in Table 1, along with two additional matrisome categories as collagens and ECM regulator which include genes pertinent to phenotypic observations. Upregulated genes have been classified according to the GO categories all of which are included in the structural constituent of cuticle category. *rol-6*, *rol-4* and *sqt-1* genes are involved in similar biological processes related to collagen function yet only *rol-6* mutants show amelioration of Rol phenotype. *rol-8* and *rol-9* are non-cuticular genes where *rol-8* is a collagen and *rol-9* is molting protease inhibitor^29^. *rol-8* mutant does not respond to ketamine treatment however *rol-9* mutants despite their non-collagen classification responds to ketamine-treatment. One interesting fact about these six mutants analysed for their response to ketamine in terms of rolling movement is that *rol-6* and *rol-9* which are responsive to ketamine are right-handed rollers whereas *rol-4*, *rol-8*, *rol-1 and sqt-1* are left-handed rollers. *rol-1* was not detected in the differentially regulated gene list. There are 173 predicted cuticular collagens in *C. elegans* 55 of which are upregulated as shown in Table 1 corresponding to approximately 30% of the entire cuticular collagen genes^30^.

**Table 1:** RNA-seq identifies ketamine-treatment upregulates 30% of the cuticular collagens.

Matrisome category	Upregulated in ketamine-treated wild-type animals	Structural constituent of cuticle GO:0042302	Collagen trimer GO:0005581	Cuticle development GO:0042335	Molting cycle GO:0042303	Extracellular region GO:0005576
Cuticular collagens	*rol-6, rol-4, bli-1, sqt-1, dpy-2, dpy-5, dpy-7, dpy-9, lon-3*	✓	✓	✓	✓	
	*bli-2, bli-6, sqt-2, dpy-3, dpy-4, dpy-8,*	✓		✓	✓	
	*lon-3, col-36, col-39, col-90, col-91*	✓	✓			
	*col-14*	✓	✓	✓		✓
	*col-115*		✓			
	*dpy-9, col-41, col-133, col-138, col-120, col-38, col-176, col-48, col-165, col-167, col-121, col-130, col-73, col-97, col-74, col-95, col-146, col-65, col-77, col-168, col-104, col-175, col-170, col-156, col-60, col-58, col-50, col-63, col-109, col-173, col-125, col-88, col-169, col-162*	✓				
Collagen	*rol-8*	✓				
	*col-17*	✓	✓			✓
ECM regulator	*rol-9*	✓				

Enrichment profiling of the ketamine-responsive downregulated genes did not have any collagen or ECM related genes (Supplementary Table 1c). The top 5 GO categories for downregulated genes are ATP synthesis coupled electron transport (GO:0042773), respirasome (GO:0070469), NADH dehydrogenase complex (GO:0030964), transporter activity (GO:0005215) and dephosphorylation (GO:0016311) (Supplementary Table 1d).

Consistent with our phenotypic observations, RNA-seq experiments identified cuticular collagens as ketamine-responsive genes some of which have been reported to be regulated via Wnt and TGF-beta pathways. Cuticular collagens upregulated upon ketamine treatment, namely *rol-6, lon-3, col-41, col-176, col-48, col-165, col-167,* has been reported to be regulated by TGF beta pathway^31,32^. Wnt-regulated ketamine-responsive collagens include *bli-1, lon-3, col-38, col-138, col-176*^33,34^. Recently, TGF-beta signaling has been implicated in antidepressant effects of ketamine where reduced expressions of Tgfb1 and its receptors in a rodent model of depression was ameliorated by ketamine administration^35^. TGF-β1 is proposed as a novel therapeutic for depression by the virtue of its’ psychotomimetic side-effect free characteristic as opposed to ketamine.

Another regulatory factor, BLMP-1 which is the homolog of mammalian PRDM1/BLIMP1, was reported to regulate a group of collagen genes some of which overlap with our ketamine-responsive gene list, namely *rol-6, rol-8, bli-1, dpy-4, dpy-5 dpy-7, dpy-8, dpy-9*^36,37^. Earlier reports have already demonstrated a modulatory role for ketamine in Wnt and TGF-beta pathways in mammalian systems^35,38^. However, BLIMP-1/BLMP-1 transcriptional regulator has not reported with ketamine-responsive roles before. Our findings identify BLIMP-1/BLMP-1 as a putative molecular target for ECM modulatory effects of ketamine. In addition to cuticular collagens targeted by BLIMP-1/BLMP-1, upregulated ketamine-responsive gene list includes a direct target of BLIMP-1/BLMP-1 that is *bed-3* which is a transcription factor with roles in molting cycle (Supplementary Table 1a)^39^. We propose that ketamine modulates ECM components in part via orchestration of Wnt and TGF-beta signalling pathways and regulatory network of transcription factor BLMP-1. Our findings suggest BLIMP-1/BLMP-1 might be a putative novel target for ketamine-mediated ECM modifications.

## Discussion

In this study, we aimed to decipher ketamine-mediated modifications using *C. elegans* as a model. Ketamine has been historically used for its anesthetic action and has recently been exploited as an alternative treatment for treatment-resistant depression. The anti-depressant action of ketamine infusion therapy is complex and not clear. Rodent models have pointed out alterations in the ECM of specific brain regions resulting in synaptic remodelling as one of the underlying mechanisms. Further investigation is required particularly in in vivo systems to gain broader understanding of ketamine’s actions.

We have found that the vulval epithelium cells of the ketamine-treated animals fail to fully migrate dorsally after detachment from the cuticle leading to defective invagination thus Sqv phenotype. At L4 stage, ketamine-treated animals show no gross vulval lumen abnormality. By the adult stage, ketamine-treated animals display an abnormally protruding vulva. When the animals are filled with eggs, ketamine’s effect on vulval epithelium becomes more visible. Some of the *sqv* mutants have also been observed with a weaker Sqv phenotype at L4 stage such as *sqv-5(k172)* and *sqv-5(k175)* yet these mutants display an abnormally protruding vulva at adult stage similar to our observation in the case of ketamine-treated animals^22^. Closer look into the different types of vulval cells have shown that the Sqv phenotype in ketamine-treated animals does not result from defects in cell differentiation. To date, Sqv phenotype has been described for worms defective in the GAG biosynthesis and modification^16,17,40,41^. One exception is *lin-35; spr-1* double mutant animals which carry mutations in the mammalian orthologues of Retinoblastoma (Rb) and CoREST, respectively^42^. *lin-35; spr-1* double mutants display a more severe Sqv phenotype with an abnormally compressed vulval lumen at the L4 larval stage. Despite their Sqv phenotype, *lin-35; spr-1* animals have wild-type like chondroitin levels and distribution similar to ketamine-treated animals. The initial inflation of the vulval lumen at early L4 stage is dependent on chondroitins. Vulval lumen matrix have recently been shown to contain dynamic apical ECM components filling the vulva lumen during morphogenesis^19^. After the early luminal inflation by chondroitins, the vulval lumen is shaped by the transient localisation of these apical ECM components including ZP proteins, the extracellular leucine-rich repeat only proteins and lipocalin. aECM assembly in the vulval lumen mediates the correct vulval eversion independent of chondroitin. This observation is in line with our ketamine results where initial vulval inflation is normal and vulval eversion is disrupted which is suggestive of alterations in the network of aECM factors which do not impinge on chondroitin. This is also in parallel with the wild-type like immunostaining observed in ketamine-treated animals. However, it is possible that mild modifications in the chondroitin biosynthesis and distribution which were not detectable in our study, is sufficient to induce Sqv phenotype.

Repeated subanesthetic dose of ketamine infusion therapy is currently being used in the clinic to treat major depression (MD)^1,43^. Low-dose ketamine administration ameliorates symptoms of MD which is sustained long after the drug is metabolised. One of the proposed mechanism of anti-depressant action for ketamine is the altered perineuronal net (PNN) composition as evidenced from recent studies conducted on rats^4,5^. PNNs are composed of ECM molecules including chondroitin, linker proteins and hyaluronic acid. They surround specific types of neurons in the brain. ECM constitutes about one fifth of the brain’s volume and is involved in development and plasticity. The brain ECM has considerable importance for the physiology of synaptogenesis with evidence supporting their involvement in the etiology of psychiatric disorders such as major depression, schizophrenia, bipolar disorders, and autism^44,45^. ECM components hold the potential to be candidate therapeutic targets for neuropsychiatric diseases. In addition to its therapeutic use in the clinic to treat depression, ketamine has been used to generate schizophrenia model in rats by its virtue of ECM modification^10^. Therefore, ketamine administration can result in adverse side effects including schizophrenia-like phenotypes and dissociation limiting its therapeutic use. As opposed to findings in the rodent models on remodelling of PNN mainly via alterations in the chondroitin, we have found that ketamine-induced aECM alterations resulting in vulval invagination defect is independent of chondroitin. The vulval epithelial phenotype upon ketamine-treatment may not include chondroitin however, it is possible that chondroitin localised in the neural ECM is modified which was not investigated in this study.

*Caenorhabditis elegans* shares similar set of signalling molecules as its mammalian counterparts and has been creatively used as a model organism to decipher molecular mechanisms of antipsychotic drugs^46^. One strategy to investigate novel targets of antipsychotic drugs in *C. elegans* was to exploit the developmental delay induced by these drugs which was in part due to decreased pharyngeal pumping rate^47^. Similar to these drugs, ketamine-treatment induced developmental delay and inhibition of pharyngeal pumping. Animals carrying mutations in known ketamine receptors in the worm, that is RyR and NMDA receptors, namely *unc-68* and *nmr-1* showed delayed development (data not shown) and Sqv phenotype implicating a mechanism of action for ketamine independent of its anesthetic action. There are very few studies conducted on ketamine’s action in *C. elegans* and high doses of ketamine is administered in these studies, up to 30 mM as opposed to 2.5 mM in our study^20^. To our knowledge, this is the first study to show ECM modulatory role for ketamine in the worm. The observations presented in our paper is in the long-lasting category as opposed to ketamine’s transient anesthetic action. Ketamine plays NMDA-independent roles in several cellular processes and mechanism underlying the long-lasting effects of ketamine is not well understood^48,49^. In a glioma cell line model where NMDA receptor was knocked down, ketamine was still able to exert antidepressant effects^50^. Given that *nmr-1*; *unc-68* double mutants display Sqv phenotype similar to single mutants, ketamine’s long-lasting effect is likely to be independent of its NMDA blockade. Through which receptors ketamine functions to modify ECM in the worm requires further investigation. Studies performed on rodent models and cell lines have shown that the predominant site of action for ketamine is neurons due to expression pattern of ketamine receptors. However, recent studies demonstrated extraneuronal sites for ketamine^51^. Our findings suggest that ketamine is likely to act nonautonomously to induce vulval and cuticular changes.

Chondroitin biosynthesis mutants *sqv-5* ad *sqv-7* display HSN migration defects along with their characteristic Sqv phenotype. Genes involved in correct HSN migration at adult stage include epidermal microRNA *mir-79* and *hst-2* which function in maintenance of heparan sulfate homeostasis^23,52^. However, these mutants do not display Sqv phenotype. Ketamine-treated animals show HSN migration defect at adulthood but not at the beginning of development (L1). Modifications of the extracellular matrix later in development may account for this difference. Ketamine may act nonautonomously to induce HSN migration defects. Amelioration of roller phenotype in ketamine-treated *rol-6* and *rol-9* mutants suggests that ketamine modulates multiple components of aECM. Collagen defect is the underlying cause for the movement of worms in a rolling fashion. Consistent with our phenotypic observations, RNA-seq experiments identified a group of ketamine-responsive cuticular collagens corresponding to 30% of the entire cuticular collagen genes in *C.elegans*. Upregulated gene list included all the *roller* mutants assessed for their movement defect except *rol-1*. Upregulation of majority of the cuticular collagens suggests a complex network for ketamine’s mechanism of action. We observed that the *roller* mutants which do not respond to ketamine are all left-handed rollers, namely *rol-4, rol-1, rol-8 and sqt-1*. The right-handed rollers *rol-6* and *rol-9* show enhancement in their movement suggesting ketamine modulates specific class of collagen complexes. The handedness of the roller phenotype stems from specific mutations which has an effect on the processing and trimerization of pro-collagens^53^. Cuticle development is a complex process. There is an extensive interaction network within the cuticular collagens which is not well understood. Mutations that induce left and right body-handedness has been described however the underlying mechanism remains obscure. Further research with the use of ketamine may shed light on possible mechanism for handed rolling behaviour. The collagen-related defects can be dose-dependent which might account for the *rol-4* mutants’ subtle movement improvement which did not reach significant levels^54^.

Enrichment analysis of differentially regulated ketamine-responsive genes identified collagens some of which have been reported to be regulated via Wnt and TGF-beta pathways^31–34^. Previous reports in mammalian systems have demonstrated a modulatory role for ketamine in Wnt and TGF-beta pathways^35,38^. Our enrichment analysis has shown that BLIMP-1/BLMP-1 might be a putative molecular target for ECM modulatory effects of ketamine by the virtue of overlapping collagens with altered expression. BLIMP-1/BLMP-1 functions both as a transcriptional repressor and a transcriptional activator and was reported to positively regulate *bed-3* which showed increased expression levels in the ketamine-treated animals suggesting ketamine may upregulate collagen and *bed-3* expression via BLIMP-1/BLMP-1-mediated gene regulation^39^. One study conducted on a cell line model inferred that ketamine might inhibit Blimp1 expression^55^. Our findings suggest that BLIMP-1/BLMP-1 might be a putative novel molecular target for ECM modulatory effects of ketamine. Given that ketamine-regulated factors hold the potential to be therapeutic targets for treatment of treatment-resistant depression, using *C. elegans* as a model to decipher ketamine-mediated alterations may provide insights into its’ roles in the mammalian systems.

A limitation of this study include the types of tissues examined for ketamine-induced modifications. Although we demonstrated a neuronal migration defect, further studies focused on neural ECM are required. Ketamine may not act on the chondroitin in the context of vulva, however which aECM molecules are altered by ketamine-treatment remains to be deciphered. Further evidence is required whether these findings in *C. elegans* can be extended to the mammalian systems. In conclusion, we propose *C. elegans* as a putative animal model to study ketamine-mediated ECM alterations.

## Materials and Methods

### Strains and maintenance

Nematode strains were cultured on Nematode Growth Media (NGM) seeded with OP50 bacteria and maintained according to standard techniques. Experiments were performed at 25 °C unless otherwise specified. The animals used for ketamine treatment are wild-type Bristol N2. Strains carrying following transgenes or alleles were used: For visualisation of HSN neuron at L1 stage *otIs33[kal-1::GFP]* IV; for assessment of HSN migration and branching defects at adult stage *zdIs13[tph-1::GFP]* IV; for detection of vulval abnormalities *syIs49[zmp-1::GFP* + *(pMH86) dpy-20(* +*)]* IV, *syIs51[cdh-3::CFP* + *unc-199(* +*)]* V; *syIs55 [ceh-2::YFP* + *unc-119(* +*)]* X, *ncIs13[ajm-1::GFP]* and *ccIs4251 [(pSAK2) myo-3p::GFP::LacZ::NLS* + *(pSAK4) myo-3p::mitochondrial GFP* + *dpy-20(* +*)]* I; to test whether sqv phenotype can be induced in mutants with defective NMDA and RyR receptors *nmr-1(ak4)* II and *unc-68(e540)* V; to evaluate roller phenotype amelioration *rol-1(e91)* II, *rol-4(sc8)* V, *rol-6(e187)* II, *rol-8(sc8)* II, *rol-9(sc148)* V and *sqt-1(sc13)* II.

### Ketamine treatment, pharyngeal pumping rate measurement and brood count

Synchronised L1 wild-type animals were grown on OP50-seeded NGM plates containing 2.5 mM ketamine and in M9 buffer as vehicle for control condition. Ketamine-treated and control animals were grown at 25 °C and all experiments were carried out at this temperature. Ketamine concentrations of 1 mM, 2.5 mM, 5 mM, 10 mM were used where higher concentrations induced high toxicity and severe developmental delay (data not shown). The 2.5 mM concentration was selected on the basis of strength of the phenotypes. Developmental delay in *C. elegans* is common when exposed to various drugs including anesthetics. 2.5 mM concentration allowed to carry out experiments without severe developmental delay and with observable phenotypes. Pharyngeal pumping rate was determined on young adult animals for three 20-s periods. One pump is defined as a single cycle of contraction and relaxation of the terminal bulb muscles. At least 10 animals were used for per three independent measurements.

Ketamine-treated and control animals were transferred to individual bacteria-seeded NGM plates with a single L4 stage animal per plate and grown at 25 °C. Animals were transferred to new plates every 24 h until they stopped egg-laying. The number of progeny on each plate was determined with three independent experiments using 10 hermaphrodites per condition.

### Immunofluorescence analysis

The immunofluorescence was performed as described previously with some modifications^56,57^. Ketamine-treated and control embryos were washed with M9 buffer and embryo pellets were fixated with 4% PFA overnight at 4 °C after four rounds of freeze-crack step in liquid nitrogen. Fixative was removed by washing in PBS containing 0.25% Triton-X (PBST). Samples were treated with 50 mU/ μl chondroitinase ABC (CSase ABC) enzyme prepared in 0.04 M Tris Acetate, pH 8. As per negative control, samples were incubated in 0.04 M Tris buffer without the CSase ABC. The incubation period for with and without CSase ABC treatment was 3 h at 37 °C. Enzyme was heat inactivated at 95 °C for 5 min. For blocking, samples were treated with 10% donkey serum for one hour at room temperature. Primary antibody incubation was performed using anti-chondroitin antibody (anti-proteoglycan ΔDi-0S antibody 1B5, 1:20; Seikagaku) at 4 °C overnight. For the secondary antibody, donkey anti-mouse Alexa 488 (Life Technologies) was used with a dilution of 1:250. VectaShield mounting medium with 49.6-diamidino-2-phenylindole (DAPI) was used to mount embryos on the microscope slides.

### Real-time RT-PCR

Transcript levels were measured as previously reported^58^. Control and ketamine-treated animals were synchronized as described above. Worms were collected at the stage of adult day 1 and washed in ice-cold M9 buffer twice. Supernatant was removed and TRIzol reagent was added to the worm pellet with a ratio of 1:2. Animals were frozen in liquid nitrogen with three cycles of freeze–thaw. RNA extraction was carried out using Direct-zol RNA kit (Zymo Research) as per manufacturer’s instructions. Briefly, worm pellet lysed in TRIzol was spun for 1 min at 12.000 g. Supernatant was mixed with absolute ethanol (1:2) and loaded onto spin columns. After washing steps, in-column DNase I treatment was performed. cDNA was synthesized from 4–10 μg total RNA using the Transcriptor High Fidelity cDNA Synthesis Kit (Roche). Real-time PCR was conducted using Roche LightCycler 480 II. *sqv-7* and *sqv-5* transcript levels were analyzed using ∆∆Ct method with *ubc-2* as a normalization control.

### RNA sequencing

Synchronised L1 wild-type animals were distributed over NGM plates containing 2.5 mM ketamine or in M9 buffer as vehicle and grown at 25 °C. RNA was isolated from whole worms at the stage of adult day 1 as described above. RNA Integrity Number (RIN) quality score was assessed using a Bioanalyzer 2100. mRNA libraries were constructed using using the Illumina Tru-Seq Stranded mRNA Library Construction Kit. 150-bp paired-end reads were generated on an Illumina HiSeq 2500 machine with 70–90 million reads per sample. FASTQ output files were aligned to the *C. elegans* reference genome (WBcel235) using Hisat2 v2.0.5. The mapped reads were assembled by StringTie (v1.3.3b) (Mihaela Pertea.et al. 2015). FPKM of each gene was calculated using featureCounts v1.5.0-p3. Differential expression analysis was performed using the DESeq2R package (1.20.0). Genes with at least a two-fold difference at an FDR < 0.05 were assigned as differentially expressed. Gene Ontology (GO) enrichment analysis of differentially expressed genes were implemented by the WormBase Enrichment Suite based on the WS262 WormBase release. Annotation and visualization of the gene set enrichment data was performed using WormCat 2.0.

### Microscopy

For image acquisition Leica DMI1 inverted microscope was used. Sqv phenotype was scored under the dissecting microscope (Leica M165 FC).

### Statistics

Statistical analysis was performed using GraphPad Prism Version 9.3.1. For all analysis α-level is 0.05.

## Supplementary Information

Supplementary Legends.

Supplementary Figure 1.

Supplementary Table 1.

Supplementary Information 1.

Supplementary Information 2.

Supplementary Information 3.

Supplementary Information 4.

## Data Availability

RNA-seq datasets have been deposited at NCBI Gene Expression Omnibus under the record GSE215256.
