# Supplementary material for: Ketamine induces apical extracellular matrix modifications in Caenorhabditis elegans
Source: Sci Rep. 2022 Dec 21;12:22122. doi: 10.1038/s41598-022-24632-5 (PMC9772317; doi:10.1038/s41598-022-24632-5)
Supplement: Supplementary file 1 — Supplementary Legends. [file 41598_2022_24632_MOESM1_ESM.docx]

**Supplementary Figure 1.** The cuticle structure of the *rol-9* mutants is ameliorated via ketamine exposure **(a)** *rol-9(sc148)* mutant expressing *myo-3::GFP* reporter is shown. Arrows show the position of the helical turns. **(b)** Expression pattern of ketamine-treated *rol-9(sc148)* mutant carrying *myo-3::GFP* transgene is depicted. Vulva is marked with an asterisk. Anterior is to the left. Scale bar shows 75 micron for all panels.

**Supplementary Table 1.** Differentailly regulated ketamine-responsive genes in wild-type animals are listed. **(a)** Upregulated genes are indicated. **(b)** Gene Ontology analysis performed on differentially upregulated gene list is shown. **(c)** Downregulated genes and **(d)** Gene Ontology analysis for downregulated genes are listed. Genes with at least a two-fold difference at an FDR  <  0.05 were assigned as differentially expressed.

**Supplementary Video 1. (a)** *rol-6* mutant treated with vehicle and **(b)** ketamine is shown

**Supplementary Video 2. (a)** *rol-9* mutant treated with vehicle and **(b)** ketamine is shown.
