# Supplementary figures and images for: Ketamine induces apical extracellular matrix modifications in Caenorhabditis elegans
Source: Sci Rep. 2022 Dec 21;12:22122. doi: 10.1038/s41598-022-24632-5 (PMC9772317; doi:10.1038/s41598-022-24632-5)

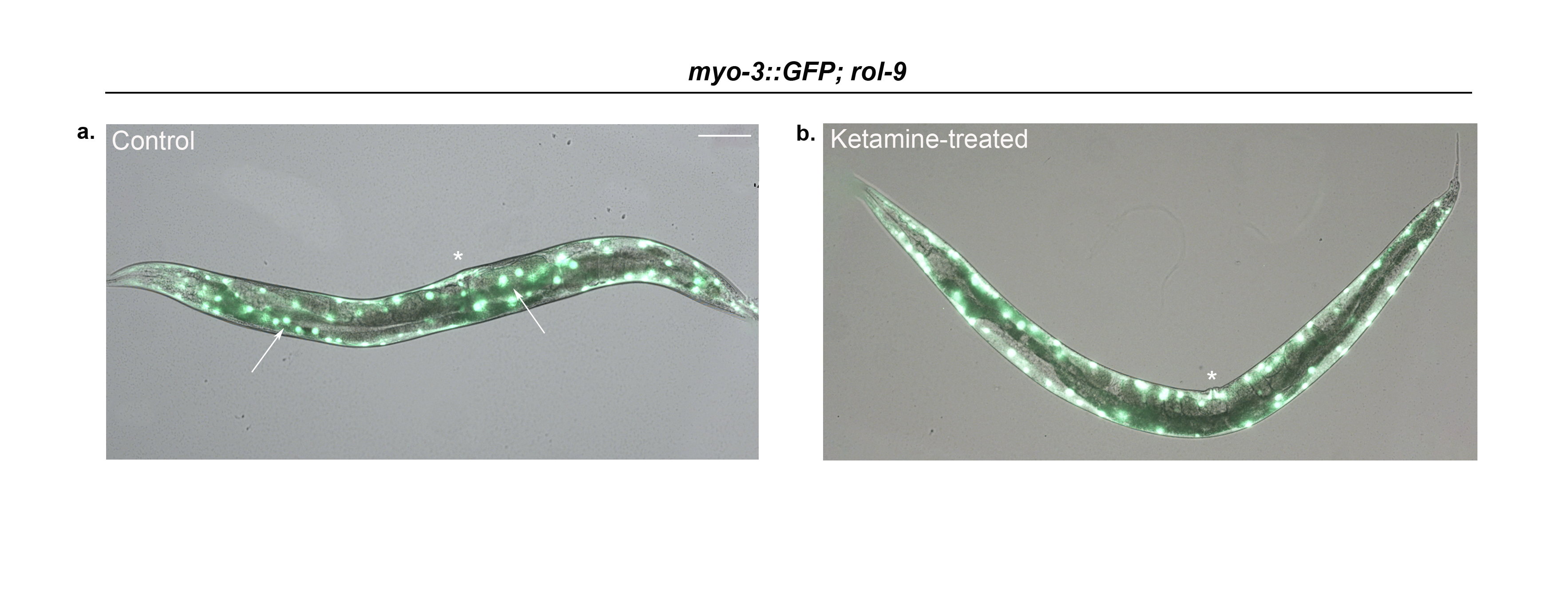

Supplement: Supplementary file 2 — Supplementary Figure 1. [file 41598_2022_24632_MOESM2_ESM.tif]
